# Preoperative communication with anesthetists via anesthesia service platform (ASP) helps alleviate patients’ preoperative anxiety

**DOI:** 10.1038/s41598-020-74697-3

**Published:** 2020-10-30

**Authors:** Fei Peng, Tao Peng, Qiange Yang, Meihan Liu, Guangxiang Chen, Maohua Wang

**Affiliations:** 1 Department of Anesthesiology, The Affiliated Hospital of Southwest Medical University, Luzhou, Sichuan People’s Republic of China; 2 Department of Radiology, The Affiliated Hospital of Southwest Medical University, Luzhou, Sichuan People’s Republic of China; 3 Southwest Medical University, Luzhou, People’s Republic of China

**Keywords:** Health care, Medical research

## Abstract

Female gender has been identified as one of the risk factors closely linked to perioperative anxiety and a lower level of satisfaction. A successful preoperative anesthesia education may improve such negative outcomes. The aim of this study was to investigate whether preoperative anesthesia education via an Anesthesia Service Platform (ASP) could reduce the anxiety levels in female patients scheduled for laparoscopic cholecystectomy under general anesthesia, and accelerate rehabilitation. A total of 222 patients scheduled for elective laparoscopic cholecystectomy were randomly assigned to the control group and the ASP group. Patients’ baseline and post-intervention psychological status was measured by the State-Trait Anxiety Inventory and General Well-Being Schedule. Pain management and recovery were assessed by VAS every 12 h for 48 h after surgery; length of stay (LOS) and postoperative analgesic consumption were also assessed. Patients in the control group experienced higher anxiety levels before surgery and had longer LOS than those in the ASP group. Patients in the ASP group had a higher general well-being score; however, they suffered more pain and consumed more analgesics after surgery. ASP is effective for preventing anxiety in female patients before laparoscopic cholecystectomy, improving patients’ general well-being levels, and shortening their LOS, but negatively influences patients’ postoperative pain levels.

## Introduction

A number of patients have insufficient knowledge about anesthesia, thinking that an anesthetist’s job only consists of administering anesthetic agents into patients’ bodies immediately before the start of the operation, after which they leave for other things while the patients are sleeping. Such misunderstanding may lead to higher anxiety levels and lower satisfaction levels^1^. As a consequence, patients’ postoperative recovery may be negatively affected due to psychoendocrinologic reactions^2,3^. For instance, anxiety may increase the cortisol secretion, which is not only considered as an acute stressor but has also been observed among patients awaiting operation^4^.

Unlike the imaginable surgical procedures, anesthesia is a relatively abstract concept, which the majority of patients are not familiar with^5^. Preoperative anesthesia education before surgery should be performed for the following reasons: firstly, an informed content should be signed by the patients or their relatives, and informing patients or their relatives on the details related to performed manipulations before surgery is governed by laws and rules^6,7^; secondly, this is a good way to earn the patient’s trust, after which patients tend to be more willing to cooperate with physicians during the treatment^8,9^; last but not least, existing evidence has validated the positive effect of preoperative education, such as decreased anxiety and pain level, reduced complications and increased confidence in fighting the illness^10,11^.

A lot of efforts have been made by different researchers to successfully implement the preoperative education, which resulted in the adoption of several following methods: (1) patients are verbally communicated the routine information; (2) pamphlets, PPT, short messages, video, and audio materials have been proposed; yet, previous studies have shown that these methods don’t allow patients to have interactive communication with their attending anesthetists or the surgeons. Also, many patients lack the ability to fully understand them^12–14^; (3) preoperative education sessions provide the possibility of interactive communication with doctors, but the time cost may be unaffordable, and patients’ compliance is not guaranteed^15^.

Previous studies investigating the aforementioned methods adopted for preoperative education reported some inconsistent outcomes. Many studies concluded that it was well worth conducting preoperative education as it could make patients experience less pain and less anxiety after surgery^2,12,13^, whereas, others reported the opposite outcomes^8,16^.

It has been demonstrated that several risk factors may have a negative influence on patients’ postoperative experience. For instance, female patients, especially those with higher educational background and younger age, have been reported to have lower satisfaction levels and higher anxiety levels after surgery^17,18^. A study showed that female patients experienced a lower quality of mental health situation, more tiredness, reduced quality of life, and higher morbidity and mortality when compared to male patients after coronary artery bypass grafting surgery^19^. Female patients are susceptible to several distressing complications and discomfort after laparoscopic surgery^20,21^.

Accordingly, the aim of this study was to investigate whether preoperative interactive communication with anesthetists via an Anesthesia Service Platform (ASP) before laparoscopic cholecystectomy under general anesthesia could reduce anxiety levels in female patients and accelerate rehabilitation.

## Materials and methods

All methods were performed in accordance with the CONSORT 2010 guidelines. The CONSORT 2010 Checklist was presented as supplementary material. The study protocol was presented as supplementary material.

### Registration and ethic approval

Our study protocol was approved by the Ethics Committee of the Affiliated Hospital of Southwest Medical University (Approval No. KY2019177) and registered on the Chinese Clinical Trial Registry on 20th January 2020 (https://www.chictr.org.cn, registration number: Chi-CTR-2000029253).

### Participants

Previous studies have reported that a change of 5 scores in the State-Trait Inventory score may be clinically relevant^22^; thus, such a change was detected with 95% power with α error of 0.05. Therefore, a minimum sample size of 111 patients in each group was enrolled between February and May 2020. Informed consent was obtained from all participants or their relatives. Patients’ actual written informed consent were not enclosed with our article because this in itself breaches the patients’ confidentiality. The informed contents are held in the patients’ hospital records.

Patients who met the following criteria were included: (1) female patients aged 20–60 years old; (2) with ASA status of I–II; (3) scheduled for elective laparoscopic cholecystectomy under general anesthesia.

Patients who met any of the following criteria were excluded: patients (1) suffering from any chronic illness or sight impairment; (2) with a history of psychological illness; (3) unable to read or write; (4) didn’t have a mobile phone; (5) underwent general anesthesia within 6 months; (6) with a difficult airway; (7) with reduced compliance level; (8) underwent day time surgery.

Patients were randomly assigned to two groups: the intervention group that received preoperative anesthesia education via ASP along with verbal information and the control group that received verbal information only.

The allocation sequence was generated by SPSS software Version 25.0 (SPSS Inc., Chicago, IL, USA). After a new patient was selected for surgery, the SPSS software was used to randomly generate a number within the range of 1 to 222, which was then used as the patient's sequence number. Patients with a sequence number of 1 to 111 were allocated to the intervention group, while those with a sequence number of 112 to 222 were allocated to the control group.

### The anesthesia service platform (ASP)

The ASP was developed through WeChat (Version 7.0.8, Tencent Technology Co.Ltd, Beijing)—the most widely used social software in China. Patients could communicate with their attending anesthetists at any place by following the Anesthesia Service Platform official account before surgery. Their attending anesthetists individualized the preoperative education information according to patients’ questions. Any questions about anesthesia, including preoperative preparation, knowledge about anesthetics or anesthetic manipulations and postoperative pain management were welcome and were carefully answered through the ASP official account.

### Treatment for the control group

The preoperative interview was completed a day before surgery by the same anesthetist (Maohua Wang) in order to avoid inconsistent verbal information. The anesthetist had a positive attitude so as not to affect patients’ psychological status in a negative way. The information listed on the anesthesia informed contents was verbally delivered to patients in both groups in a private room.

### Treatment for the ASP group

After the preoperative interview was completed by the same anesthetist (Maohua Wang), patients in the ASP group were asked to follow the Anesthesia Service Platform official account. Immediately after the preoperative anesthesia interview, the attending anesthetist sent a video displaying the certain anesthetic approach under which the surgery was performed. Patients were also informed that any questions about anesthesia, including preoperative preparation, knowledge about anesthetics or anesthetic manipulations, and postoperative pain management, are welcome and will be carefully answered through the ASP official account.

### Anesthesia and surgery

All patients fasted for 8 h before surgery. The intravenous access was established after patients’ arrival into the operation room by the same nurse, followed by continuous monitoring of their vital signs, including pulse oxygen saturation, five-lead electrocardiogram, heart rate, and bispectral index (BIS). Noninvasive blood pressure was recorded every 5 min. All patients were premedicated with penehyclidine hydrochloride 0.3 mg (Chengdu List Pharmaceutical Co., Ltd, Chengdu, China) intravenously and midazolam 0.04 mg/kg (Jiangsu Nhwa Pharmaceutical Co., Ltd, Xuzhou, China) orally 30 min before surgery. For anesthesia induction, propofol 2 mg/kg (Xi’an LiBang Pharmaceutical Co., Ltd, Xi’an, China) and sufentanil 0.3 µg/kg (Yichang Renfu Pharmaceutical Co., Ltd, Yichang, China) were intravenously injected. Insertion of the laryngeal mask airway was facilitated by intravenous administration of cis-atracurium 0.2 mg/kg (Jiangsu Hengrui Pharmaceutical Co., Ltd, Lianyungang, China). After the establishment of an artificial airway, volume-controlled ventilation with a tidal volume of 8 mL/kg was initiated. The respiratory rates were adjusted to stabilize the end-tidal CO_2_ partial pressure close to 4.7 kPa. Anesthesia was maintained by Propofol 4–12 mg/kg/h and remifentanil (Yichang Renfu Pharmaceutical Co., Ltd, Yichang, China) 0.1–0.2 µg/kg/min to stabilize BIS scores at 40–60, heart rate, and noninvasive mean blood pressure within 20% of their basic levels. A bolus of cis-atracurium was administered if electromyographic signal appeared. All patients were wheeled to the Post-anesthesia Care Unit (PACU).

The same surgeon and his assistant performed all the surgeries; thus, it was possible to eliminate the potential impact of the surgeon factor on the results.

### Measures

#### Baseline data

Patients’ baseline data, including age, BMI, previous anesthesia, educational background, duration of anesthesia, and duration of operation, were independently collected by two authors (Fei Peng and Tao Peng), while any discrepancies were solved by a third one (Maohua Wang).

#### Patients’ preoperative anxiety

After the preoperative anesthesia interview, patients in the control group were asked to complete the State-Trait Anxiety Inventory (STAI) scale to measure their preoperative anxiety. This scale was developed by Spielberger et al.^23^, and has been shown to be a reliable and valid instrument^24^. The scale comprises two separate parts (State Anxiety Scale and Trait Anxiety Scale), each one containing 20 items. The State Anxiety Scale is mainly used to reflect patients’ immediate or recent feelings of fear, tension, anxiety, and neuroticism at a particular moment. Patients were asked to describe their distinctive feelings through the Trait Anxiety Scale. Final scores ranged from 20 to 80, with higher scores indicating lower levels of anxiety.

In the ASP group, all patients were required to fulfill the STAI Scale before their next ASP official account, and only those who had interactive communication with the attending anesthetist (Maohua Wang) were sent the SAI Scale, and they were required to return the completed scale to the anesthetist on ASP before surgery.

#### Patients’ general well-being level

Patients’ general well-being level was measured by the General Well-Being Schedule (GBWS) by the same anesthetist (Fei Peng) shortly before their discharge. The schedule consists of 33 items covering 6 parts: anxiety, depression, freedom from a health concern, vitality, life satisfaction, and emotional-behavioral control, with a maximum score of 110, where higher scores indicate a higher level of general well-being. Good internal consistency was observed in GWBS score (Cronbach’s α 0.90 to 0.94)^25^. This instrument has been proved valid and reliable by several large clinical and epidemiological studies^26,27^.

#### Postoperative pain management

All patients’ postoperative VAS scores were recorded every 12 h for 2 days after surgery by the same anesthetist (Fei Peng) via a 10-cm-length ruler. Patients were instructed to report their pain level via the VAS ruler immediately before the first assessment, where higher scores suggested higher pain levels. The nurses were told not to awake patients when they were sleeping.

Patient-controlled analgesia (PCA) was not allowed since certain rules decree that PCA should not be administered in order to limit the medical costs of patients undergoing laparoscopic cholecystectomy. As a result, an individualized dose of dezocine injection was administered if patients suffered from intolerable pain after surgery. The amount of dezocine administered was documented at the same time when the VAS scores were recorded.

#### Length of stay (LOS)

LOS, which was obtained from nurse records, was defined as the period between the end of the surgery and discharge.

#### The most frequently asked questions (FAQs)

We documented the top 5 most frequently asked questions in the patients’ interactive communication with their attending anesthetist.

#### Postoperative complications

According to the Clavien-Dindo classification system^28^, the severity of postoperative complications was graded, and it was used as the outcome variable to explore the independent influencing factors of the severity of postoperative complications. According to the Clavien-Dindo grading System, the postoperative complications were classified as follows: Grade I, a drug intervention is not needed. The complications are not life-threatening, but appropriate antiemetics, antipyretics, analgesics, balanced electrolyte drugs, and physical therapy are allowed; Grade II, blood transfusion, and parenteral nutrition are needed; Grade III, emergency operation is needed; Grade IV, the complications are life-threatening.

### Statistical analysis

All data were independently analyzed using SPSS Version 25.0 (SPSS Inc., Chicago, IL, USA) by two authors (Fei Peng and Hong Deng), while any discrepancies were solved by a third one (Maohua Wang). Quantitative variables were described as Mean ± SD, while qualitative data were expressed as numbers and percentages. Continuous variables were compared by a two-tailed Student t-test. A Chi-square test was used to compare the two groups for qualitative variables. A* P* value < 0.05 was considered statistically significant.

## Results

**Figure 1:**
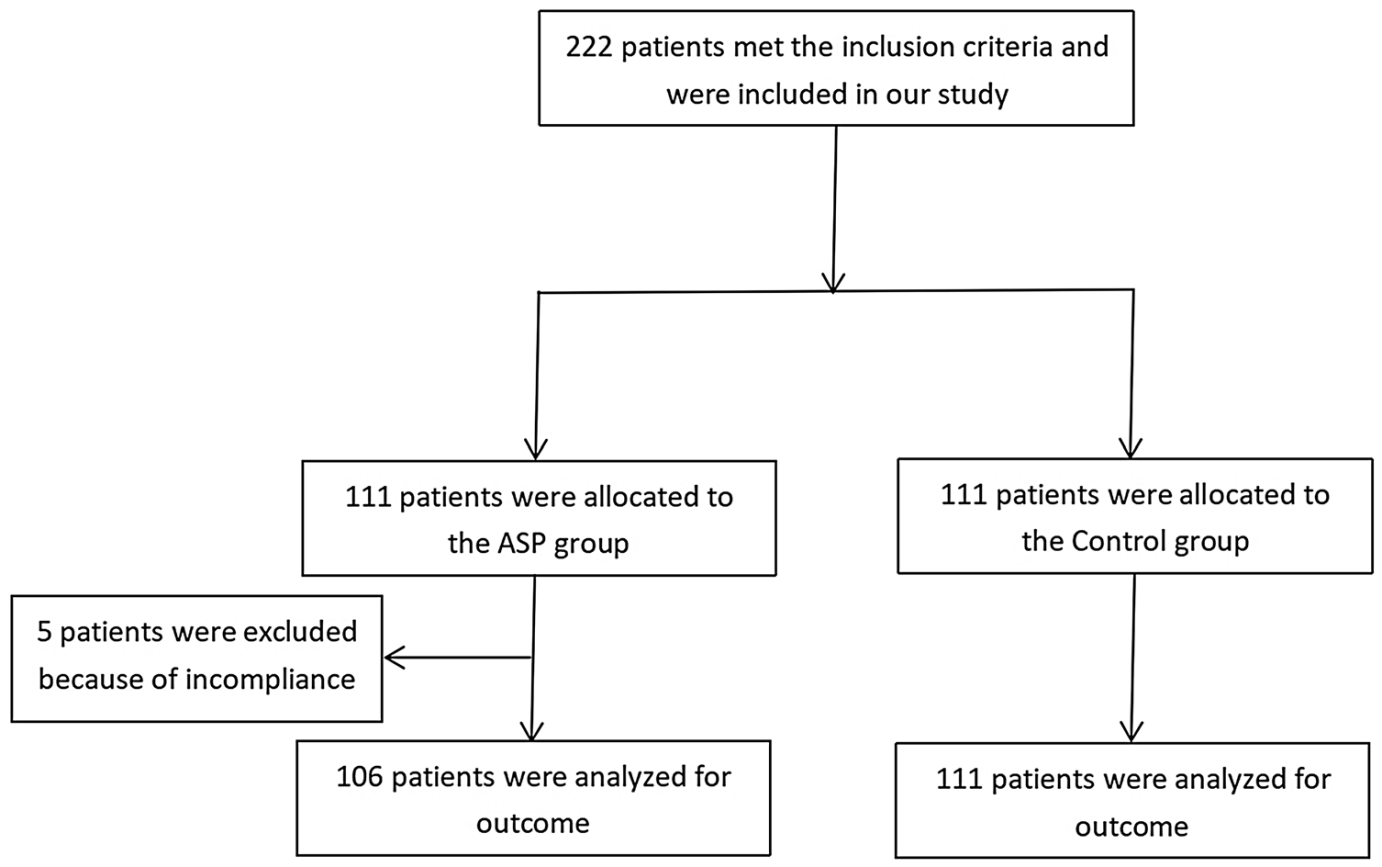
Flow diagram summarizing the experimental process.

A total of 222 patients consented to participate in our study (111 each). Finally, 217 patients were included due to incompliance of 5 patients in the ASP group (Fig. 1). Patients’ demographics are presented in Table 1. No significant difference was found in these baseline data between the ASP group and the Control group (*P* > 0.05).

**Table 1:** Demographics at baseline, no significant difference was found in these baseline data between the ASP group and the control group (*P* > 0.05).

Items	ASP group	Control group	P value
Sample size	106	111	
**Age (years)**	45.27 ± 8.67	45.15 ± 8.20	0.916
≤ 40 years (n)	30 (28.3)	33 (29.7)	0.697
> 40 years (n)	76 (71.7)	78 (70.3)	0.951
BMI (kg/m^2^)	26.44 ± 1.99	26.99 ± 1.76	0.331
Previous general anesthesia (n, %)	6, 5.7	7, 6.3	0.841
**Education background**			
High school or lower graduates	80	86	0.728
College graduates	25	25	0.853
Master or higher	1	0	0.305
**Marital status**			
Married	94	95	0.497
Single	12	16	0.497
Previous childbearing (n, %)	98, 92.5	99, 89.2	0.406
Duration of surgery (min)	46.88 ± 15.82	48.01 ± 16.35	0.560
Duration of surgery > 60 min (n)	11	14	0.606
Duration of anesthesia (min)	60.57 ± 16.22	61.77 ± 16.74	0.590

### Patients’ preoperative anxiety

**Figure 2:**
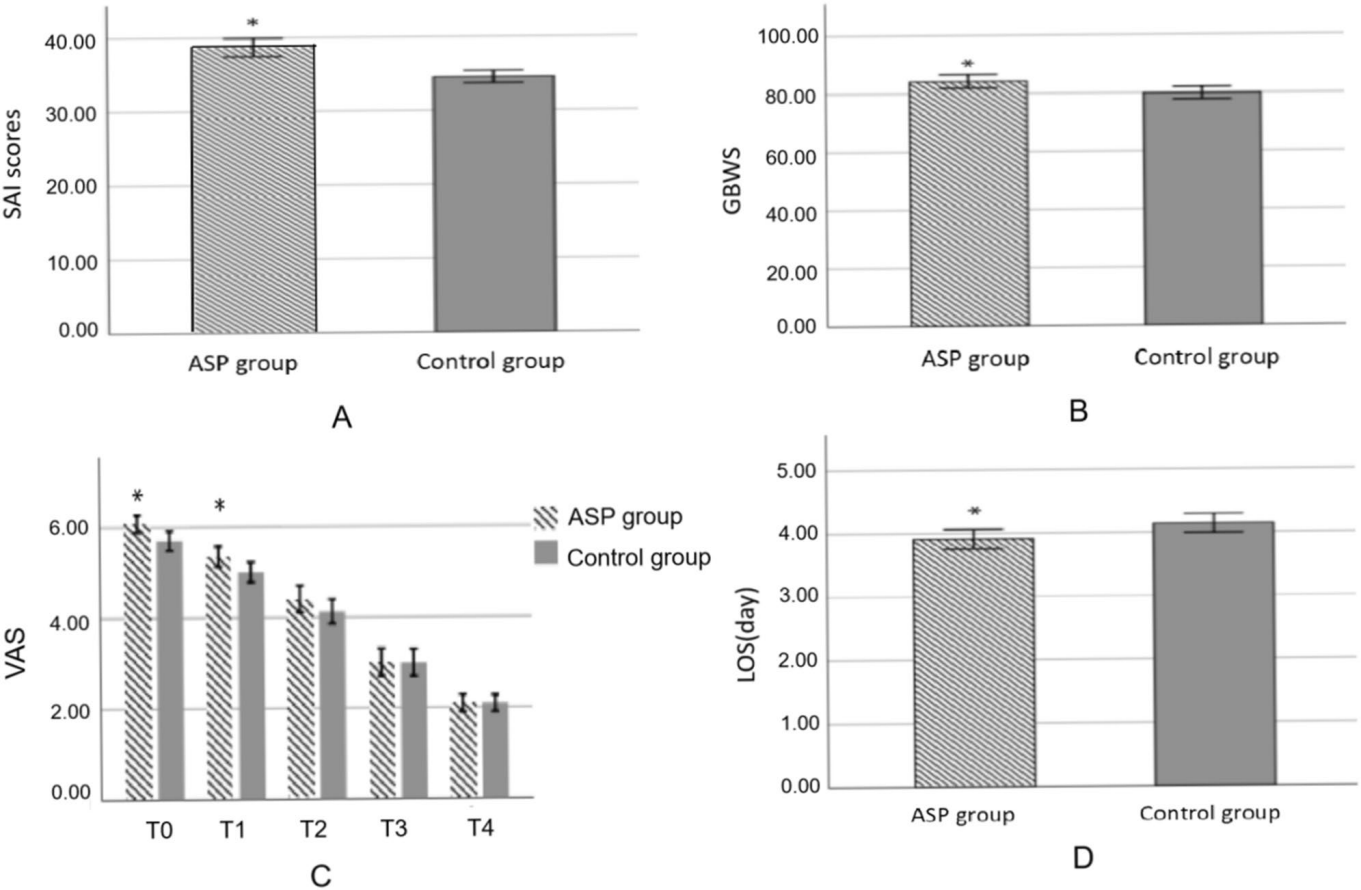
(**A**) SAI scores after interactive communication with anesthetists, patients exhibited significantly higher levels of SAI scores after interactive communication with their attending anesthetist (39.72 ± 6.27 versus 34.56 ± 4.46, *P* < 0.05); (**B**) GBWS after surgery, patients in the ASP group had higher scores in the General well-being test when compared with those in the Control group (84.25 ± 11.72 vs. 79.86 ± 11.53, *P* < 0.05); (**C**) VAS scores after surgery, Patients’ postoperative pain level measured by VAS scores showed significant differences between the two groups immediately after surgery (6.08 ± 1.01 vs. 5.70 ± 1.13, *P* < 0.05) and 12 h after surgery (5.46 ± 1.20 vs. 5.00 ± 1.18, *P* < 0.05), while there was no significant difference between the two groups at 24 h, 36 h and 48 h after surgery (4.41 ± 1.52 vs. 4.13 ± 1.43, *P* = 0.164; 3.00 ± 1.57 vs. 2.99 ± 1.59, *P* = 0.967; 2.09 ± 1.00 vs. 2.09 ± 0.98, *P* = 0.975); (**D**) Comparison of length of stay in hospital, Patients in the ASP group had shorter hospital stay compared to those in the Control group (3.91 ± 0.79 vs. 4.14 ± 0.79, *P* = 0.031).

Analysis of State Anxiety Inventory scores and Trait Anxiety Inventory scores after routine preoperative anesthesia interview showed no baseline difference between the ASP group and the Control group (36.16 ± 4.53 vs. 35.99 ± 4.54, *P* > 0.783; 34.81 ± 4.41 vs. 34.56 ± 4.46, *P* > 0.675; Figure Sa and Figure Sb). However, patients exhibited significantly higher levels of SAI scores after interactive communication with their attending anesthetist (39.72 ± 6.27 versus 34.56 ± 4.46, *P* < 0.05; Fig. 2A).

### Patients’ general well-being level

As was shown in Fig. 2B, patients in the ASP group had higher scores in the General well-being test when compared with those in the Control group (84.25 ± 11.72 vs. 79.86 ± 11.53, *P* < 0.05).

### Postoperative VAS scores

Patients’ postoperative pain level measured by VAS scores showed significant differences between the two groups immediately after surgery (6.08 ± 1.01 vs. 5.70 ± 1.13, *P* < 0.05) and 12 h after surgery (5.46 ± 1.20 vs. 5.00 ± 1.18, *P* < 0.05), while there was no significant difference between the two groups at 24 h, 36 h and 48 h after surgery (4.41 ± 1.52 vs. 4.13 ± 1.43, *P* = 0.164; 3.00 ± 1.57 vs. 2.99 ± 1.59, *P* = 0.967; 2.09 ± 1.00 vs. 2.09 ± 0.98, *P* = 0.975; Fig. 2C).

### Postoperative consumption of analgesics

Analysis of postoperative consumption of analgesic indicated that patients in the ASP group consumed less dezocine during the first 12 h after surgery than those in the Control group (Table S1a). In comparison, no significant difference was detected during the second 12 h, and the second day after surgery (Table Sb and Table Sc).

### Length of stay in hospital

Patients in the ASP group had shorter hospital stay compared to those in the Control group (3.91 ± 0.79 vs. 4.14 ± 0.79, *P* = 0.031; Fig. 2D).

### The most frequently asked questions

The five most frequently asked questions in the patients’ interactive communication with their attending anesthetist are shown in Table 2.

**Table 2:** The most frequently asked questions by patients.

Ranking	The most frequently asked questions	Percentage
Top 1	Complications of anesthesia	86.2
Top 2	Postoperative pain management	80.4
Top 3	Recovery from anesthesia	65.1
Top 4	Preoperative fasting and water deprivation	55.7
Top 5	The cost of anesthesia	32.3

### Postoperative complications

In the ASP group, 5 patients were afflicted by Grade I complications, including fever and abdominal distention compared to 6 patients in the Control group; the differences observed between the two groups were not statistically significant (Table Sd).

## Discussion

The key findings in the present study are that preoperative anesthesia education via the Anesthesia Service Platform could significantly relieve preoperative anxiety in female patients, shorten their length of stay in the hospital, and improve their general well-being during hospitalization.

Preoperative education is vital for patients and medical staff and may make patients willing to participate in the treatment process actively and positively cooperate with medics. Some of the outcomes in the present study were consistent with several previous studies, which demonstrated that a successful preoperative education might reduce anxiety, improve satisfaction, and lead to a better prognosis^29,30^. However, Anesthesia Service Platform on the mobile phone is a novel approach for preoperative anesthesia education, which enables patients to communicate with their attending anesthetist, thus obtaining individualized information. Moreover, compared with other approaches of preoperative anesthesia education, ASP makes it possible for patients to save and review important information conveyed by anesthetists.

Our results revealed that patients in the ASP group experienced more pain than those in the Control group within 12 h after surgery. Such an outcome indicated that information might sensitize patients to experience more pain, which was supported by two previous studies^16,31^. In 1995, Manyande et al.^32^ adopted an approach of imagery by which they helped to boost the patients’ confidence to deal with their stress caused by the surgery. Successfully, it helped the patients feel lower levels of pain postoperatively. Patients in the experimental group consumed fewer analgesics and experienced shorter lengths of stay in the hospital. In their conclusion, they argued that certain levels of anxiety could help patients to be well-prepared for surgery and reduce stress-related pain.

As for the details about the interactive communication between the anesthetists and their patients, over 80% of the patients cared about the complications of anesthesia and postoperative pain management. Over half of the participants in the ASP group cared about recovery from anesthesia (68.4%) and preoperative fasting and water-deprivation (55.7%). Only a minority of participants valued the costs of anesthesia (32.3%). Similarly, there was a study that reported the top three concerns to patients undergoing surgery were anesthesia safety, loss of consciousness under anesthesia, and postoperative pain management^33^. In line with our study, established evidence has demonstrated that preoperative information about anesthesia, even before the information about the surgery, could benefit patients when it comes to anxiety, prognosis, and length of stay in hospital^34^.

Although many anesthesia related questions can be answered in the anesthetic clinic or in the ward, the Anesthesia Service Platform allows for more efficient communication as anesthetists can use it to answer to patients’ questions anywhere at any time when they are free. In China, some internet or e-commerce giants, such as Baidu, Alibaba, et al., have established some online clinics in cooperation with clinicians. Whether you are a patient or a visitor, they will assist you before, during, and after your medical consultation by connecting you with professional doctors. On the other hand, they allow clinicians to work with a more flexible schedule.

Following the technological development, the mobile phone has become a trendy device that is globally used and almost an indispensable necessity. Considering the invested time, mobile fees, and clinical benefit, ASP appears as a beneficial solution for both patients and anesthetists.

This study has a few limitations. Firstly, the double blind method was not applied to reflect the real-world conditions during clinical work more closely. Although this may affect the interpretation of the outcomes, the reported results are convincing when the Anesthesia Service Platform is applied during clinical work. Secondly, the present study was consistent with several previous studies arguing that preoperative information may sensitize patients to experience more pain after surgery. Yet, the extent to which the preoperative anesthesia education could affect patients’ postoperative pain level and what’s the specific correlation between preoperative education and patients’ postoperative pain level has not been clearly elucidated and should be addressed by further studies. Last but not least, we just focused on female patients undergoing laparoscopic cholecystectomy. However, levels of anxiety may be different among patients undergoing different surgeries or among male patients; thus, further studies should focus on different types of surgeries and both genders.

## Conclusion

Interactive communication with the anesthetists via Anesthesia Service Platform before laparoscopic cholecystectomy resulted effective in relieving preoperative anxiety in female patients, improving their general well-being as well as shortening the length of their stay in hospital; however, it sensitized them to experience more pain within 12 h after surgery during which period they consumed more analgesics.

## Supplementary information

Supplementary Information 1.

Supplementary Information 2.
