# Supplementary material for: Preoperative communication with anesthetists via anesthesia service platform (ASP) helps alleviate patients’ preoperative anxiety
Source: Sci Rep. 2020 Oct 30;10:18708. doi: 10.1038/s41598-020-74697-3 (PMC7603311; doi:10.1038/s41598-020-74697-3)
Supplement: Supplementary file 1 — Supplementary Information 1. [file 41598_2020_74697_MOESM1_ESM.docx]

**Supplementary materials**

**Preoperative Communication with Anesthetists via Anesthesia Service Platform (ASP) Helps Alleviate Patients’ Preoperative**

Fei Peng1#, Tao Peng1#, Qiange Yang1, Hanmei Liu3,  Guangxiang Chen2, Maohua Wang1*
1 Department of Anesthesiology, The Affiliated Hospital of  Southwest Medical University, Luzhou, Sichuan P.R. China
2 Department of Radiology, the Affiliated Hospital of Southwest Medical University

3Southwest Medical University
# Both the authors contributed equally to this work.
*The corresponding author:Maohua Wang 
The e-mail address of the corresponding author: [wangmaohua@swmu.edu.cn](mailto:wangmaohua@swmu.edu.cn)

The telephone number of the corresponding author: +86 17313466713

***Conflicts of Interest***: We declare that there are no known conflicts of interest associated with this publication.

***Funding***: Our study was supported by Sichuan Science and Technology Department Foundation (2019YJ0692), the Joint Foundation of Luzhou Government and Southwest Medical University (2018LZXNYD-ZK02) and (2019LZXNYDJ23).

***Ethics approval***: Our study protocol was approved by the Ethics Committee of the

Affiliated Hospital of Southwest Medical University (Approval No. KY2019177) and registered on the Chinese Clinical Trial Registry (www.chictr.org.cn, registration number: Chi-CTR-2000029253).

Figure a SAI scores after routine preoperative anesthesia interview


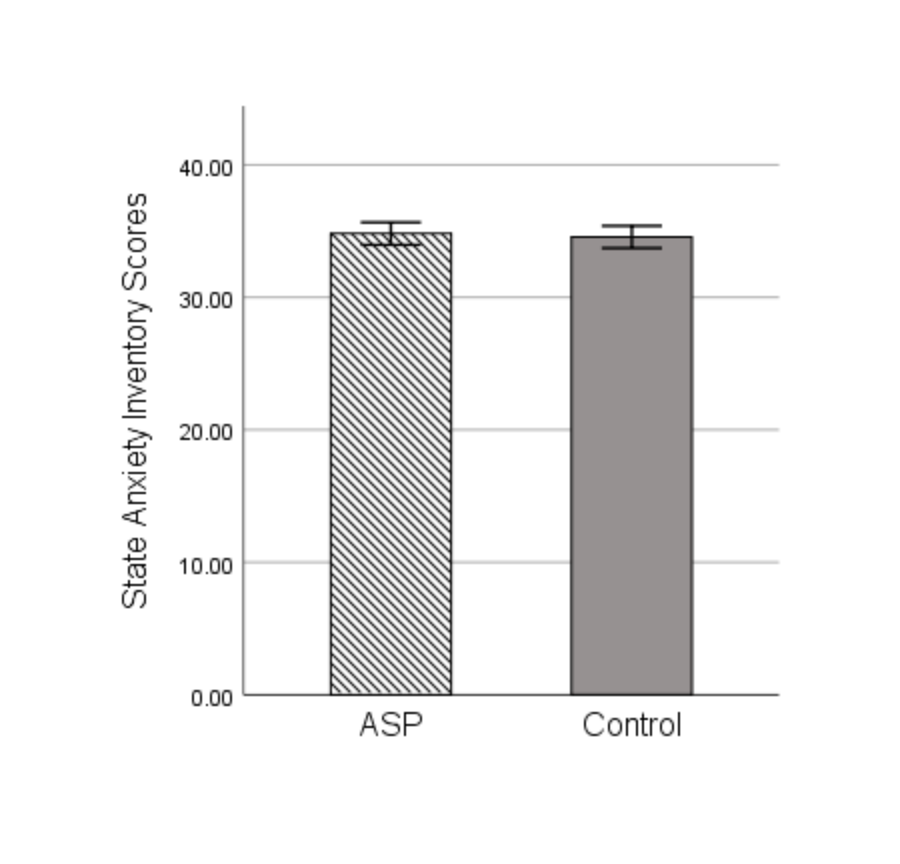


Figure b TAI scores after routine preoperative anesthesia interview


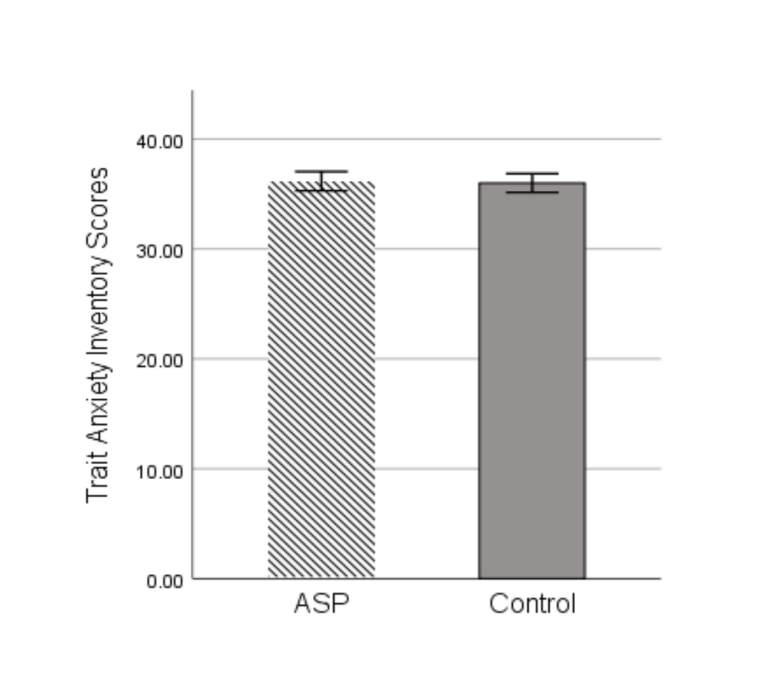


Table a Use of dezocine after surgery (The first 12h)


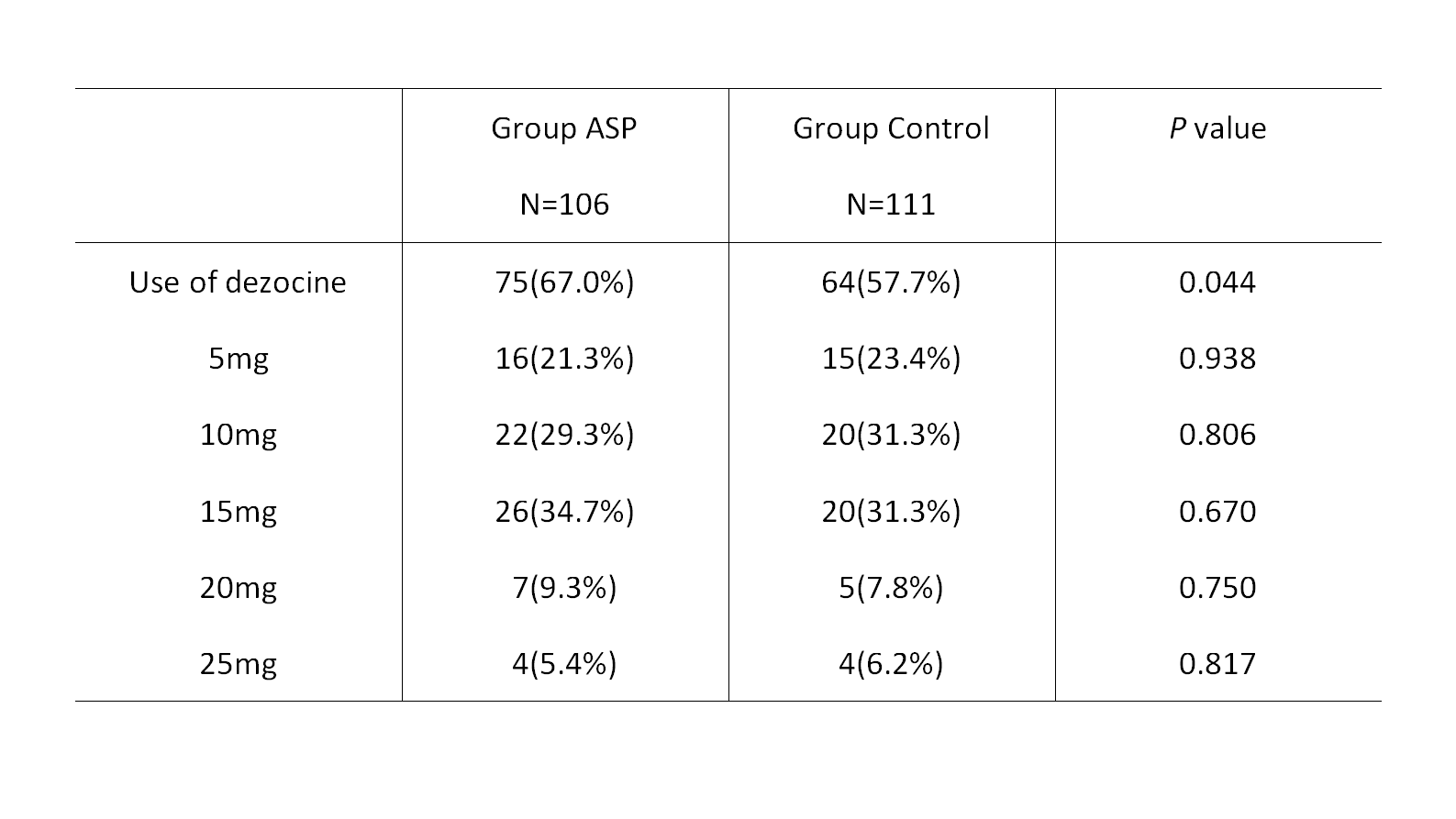


Table b Use of dezocine after surgery (The second 12h)


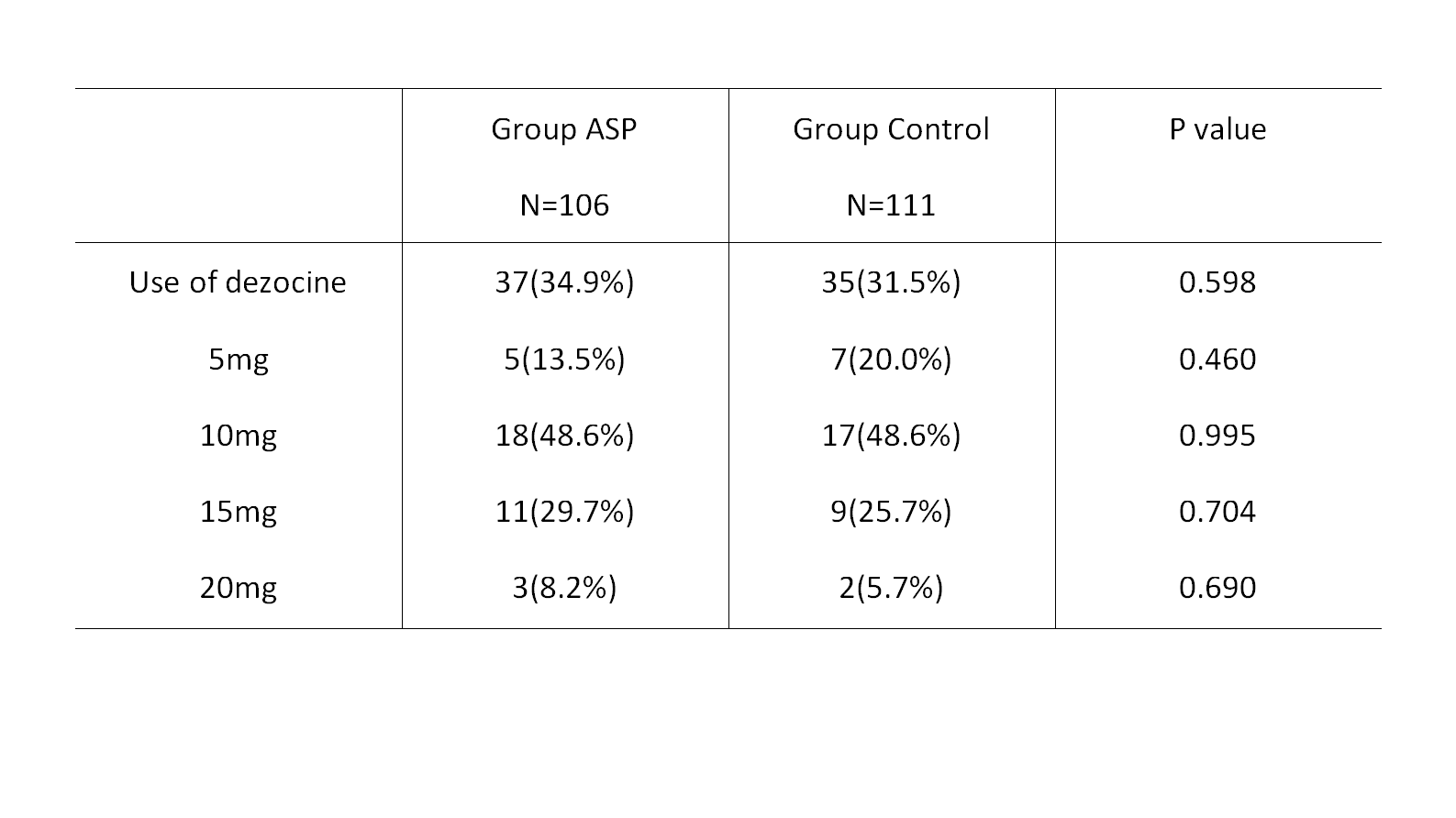


Table c Use of dezocine after surgery (The second day after surgery)


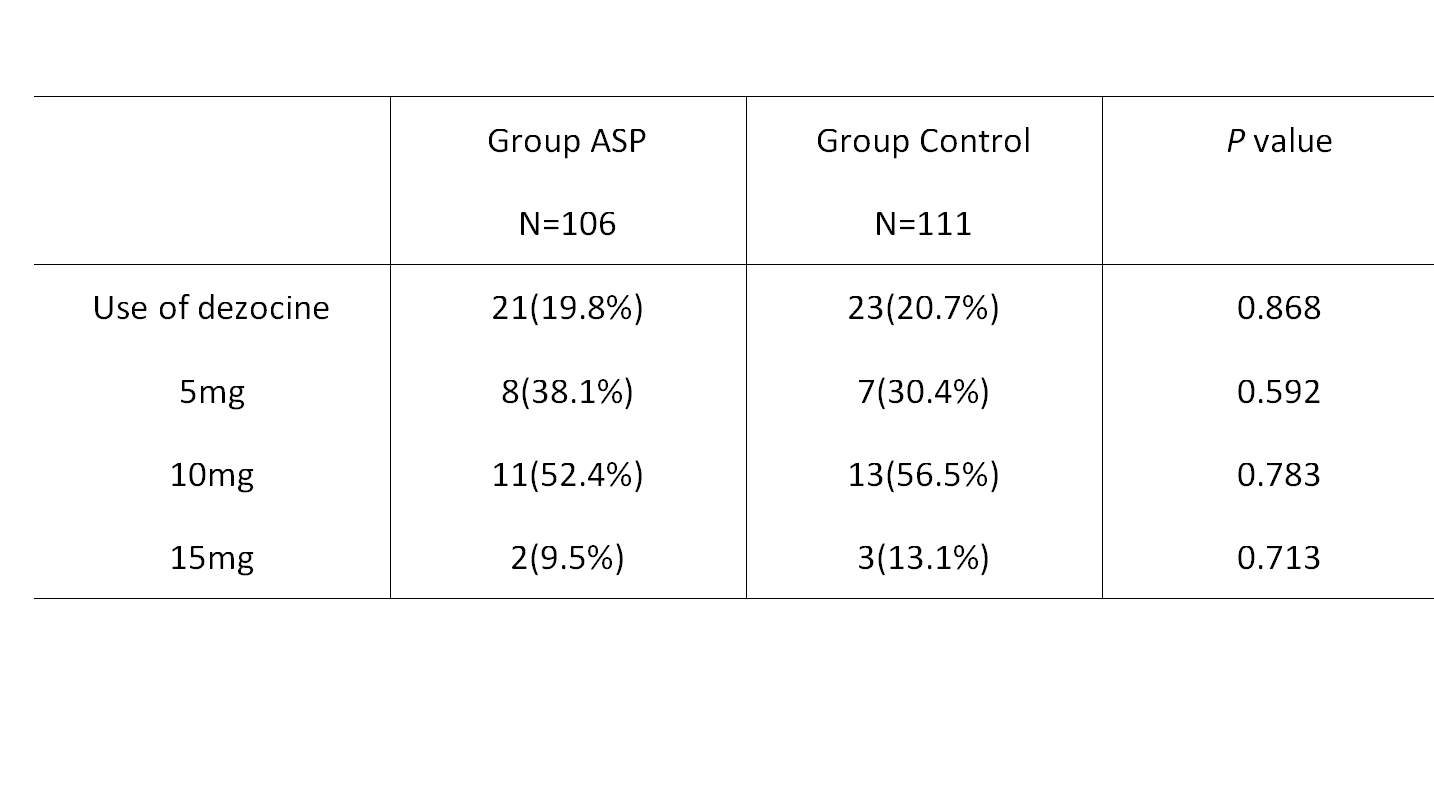


Table d Summary of postoperative complications according to the Clavien-Dindo classification system


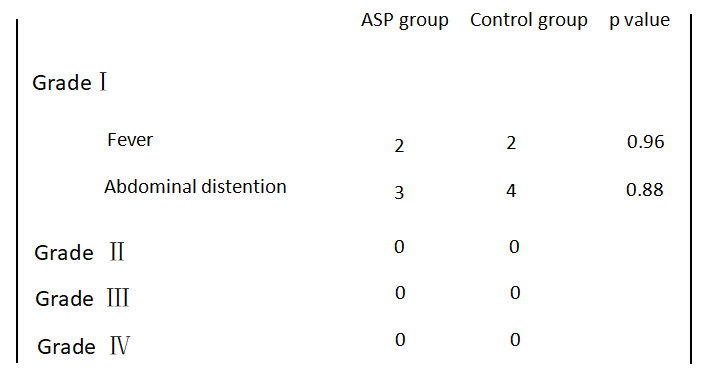


State-Anxiety Inventory

特质焦虑量表

Trait-anxiety Scale

几乎没有 有些 经常 总是如此

Never A little Often Always

1. 我感到愉快 ① ② ③ ④

1 I feel good

1. 我感到神经过敏和不安 ① ② ③ ④

2 I feel nervous and uncomfortable

1. 我感到自我满足 ① ② ③ ④

3 I feel self-satisfied

1. 我希望能像别人那样高兴 ① ② ③ ④

4 I want to be as happy as others

1. 我感到我像衰竭一样  ① ② ③ ④

5 I feel like a failure

1. 我感到很宁静 ① ② ③ ④

6 I feel very quiet

1. 我是平静的、冷静的和泰然自若的  ① ② ③ ④

7 I feel calm, cool and imperturbable

1. 我感到困难一一堆集起来，因此无法克服 ① ② ③ ④

8 I can’t overcome the difficulties which are piling up

1. 我过分忧虑一些事，实际这些事无关紧要 ① ② ③ ④

9 I worry too much about things which are really don’t

matter

1. 我是高兴的 ① ② ③ ④

10 I am glad

1. 我的思想处于混乱状态 ① ② ③ ④

11 My mind is in a state of confusion

1. 我缺乏自信心 ① ② ③ ④

12 I lack self-confidence

1. 我感到安全 ① ② ③ ④

13 I feel safe

1. 我容易做出决断 ① ② ③ ④

14 I am a decisive person

1. 我感到不合适 ① ② ③ ④

15 I don’t feel good

1. 我是满足的 ① ② ③ ④

16 I am satisfied with my life

1. 一些不重要的思想总缠绕着我，并打扰我 ① ② ③ ④

17 Unimportant thoughts always haunts me and

disturb me

1. 我产生的沮丧是如此强烈，以致我不能从 ① ② ③ ④

思想中排除它们

18 The frustration I am experiencing is so intense that

I can’t eliminate from my mind

1. 我是一个镇定的人 ① ② ③ ④

19 I am calm

20.当我考虑我目前的事情和利益时，我就陷入 ① ② ③ ④

紧张状态

20 When I consider my present fairs and interests, I fall

into tension

状态焦虑量表

State-anxiety Scale

几乎没有 有些 经常 总是如此

Never A little Often Always

1. 我感到心情平静 ① ② ③ ④

1 I feel calm

1. 我感到安全 ① ② ③ ④

2 I feel safe

1. 我是紧张的 ① ② ③ ④

3 I am nervous

1. 我感到紧张束缚 ① ② ③ ④

4 I feel nervous and restricted

1. 我感到安逸 ① ② ③ ④

5 I am at ease

1. 我感到烦乱 ① ② ③ ④

6 I feel upset

1. 我现在正烦恼，感到这种烦恼超过了可能的 ① ② ③ ④

不幸

7 I am in trouble which is unconquerable

1. 我感到满意 ① ② ③ ④

8 I feel satisfied

1. 我感到害怕 ① ② ③ ④

9 I am scared

1. 我感到舒适 ① ② ③ ④

10 I feel comfortable

1. 我有自信心 ① ② ③ ④

11 I have enough confidence toward life

1. 我觉得神经过敏 ① ② ③ ④

12 I am jittery

1. 我极度紧张不安 ① ② ③ ④

13 My tempers are on edge

1. 我优柔寡断 ① ② ③ ④

14 I am indecisive

1. 我是轻松的 ① ② ③ ④

15 I feel relaxed

1. 我感到心满意足 ① ② ③ ④

16 I feel satisfied about my life

1. 我是烦恼的 ① ② ③ ④

17 I am agonizing

1. 我感到慌乱 ① ② ③ ④

18 I am panic

1. 我感觉镇定 ① ② ③ ④

19 I am self-composed

20.我感到愉快 ① ② ③ ④

20 I feel delighted

General Well-Being Schedule

《总体幸福感量表》 Fazio编制

General Well-Being Schedule, GWBS

1. 你的总体感觉怎样?

1 How do you feel in general?

1. 好极了 B.精神很好 C.精神不错 D.精神时好时坏E.精神不好F.精神很不好

A.Excellent B.Very good C.Good D.Marginal E.Bad F.Very bad

2.你是否为自己的神经质或“神经病”感到烦恼?

2 Are you bothered by your neuroticism or mental illness?

A.极端烦恼B.相当烦恼C.有些烦恼D.很少烦恼E.一点也不烦恼

A.Absolutely upset B.Very upset C.Moderately upset D.A little upset E.Not at all

3.你是否一直牢牢地控制着自己的行为、思维、情感或感觉?

3 Do you always have a firm grip on your actions, thoughts, emotions or feelings?

A.绝对的 B.大部分是的 C.一般来说是的 D.控制得不太好 E.有些混乱 F. 非常混乱

A.Absolutely B.Mostly C.Generally D.Not so well E.Bad F.Very bad

4.你是否由于悲哀、失去信心、失望或有许多麻烦而怀疑还有任何事情值得去做?

4 Are you sad, discouraged, disappointed, or in so much trouble that you doubt there's anything worth doing?

1. 极端怀疑 B.非常怀疑 C.相当怀疑 D.有些怀疑 E.略微怀疑 F. 一点也不怀疑

A.Absolutely B.Mostly C.Very D.moderately E.A little F.Not at all

5.你是否正在受到或曾经受到任何约束、刺激或压力?

5 Are or have you been subjected to any constraints, stimuli, or pressures?

A.相当多 B.不少 C.有些 D.不多 E.没有

A.Too much B.much C.Moderately D.A little E.Not at all

6.你的生活是否幸福、满足或愉快?

6 Is your life happy, fulfilling, or enjoyable?

A.非常幸福 B.相当幸福 C.满足D.略有些不满足E.非常不满足

A.Absolutely B.Very C.Moderately D.Bad E.Very bad

7.你是否有理由怀疑自己曾经失去理智，或对行为、谈话、思维或记忆失去控制?

7 Do you have any reason to suspect that you have lost your mind or control of your actions, conversations, thoughts, or memories?

A.一点也没有 B.只有一点点 C.有些，不严重 D.有些，相当严重E.是的，非常重

A.Not at all B.A little C.Moderately D.A lot E.Too much

8. 你是否感到焦虑、担心或不安?

8 Do you feel anxious, worried, or upset?

A.极端严重B.非常严重C.相当严重D.有些 E.很少 F.无

A.Absolutely B.Much C.Very D.Moderately E.A little F.Not at all

9. 你睡醒之后是否感到头脑清晰和精力充沛?

9 Do you wake up feeling fresh and energized?

A.天天如此B.几乎天天C.相当频繁D.不多 E.很少 F.无

A.Frequently B.Always C.Often D.Sometimes E.Seldom F.Never

10.你是否因为疾病、身体的不适、疼痛或对患病的恐惧而烦恼?

10 Are you troubled by illness, discomfort, pain, or fear of illness?

A.所有时间 B.大部分时间 C.很多时间 D.有时 E.偶尔 F.无

A.Frequently B.Always C.Often D.Sometimes E.Seldom F.Never

11.每天的生活中是否充满了让你感兴趣的事情?

11 Is your life filled with things that interest you?

A.所有时间 B.大部分时间 C.很多时间 D.有时 E.偶尔 F.无

A.Frequently B.Always C.Often D.Sometimes E.Seldom F.Never

12.你是否感到沮丧和忧郁?

12 Do you feel depressed and sorrowful?

A.所有时间 B.大部分时间 C.很多时间 D.有时 E.偶尔 F.无

A.Frequently B.Always C.Often D.Sometimes E.Seldom F.Never

13.你是否情绪稳定并能把握住自己?

13 Are you emotionally stable and in control?

A.所有时间 B.大部分时间 C.很多时间 D.有时 E.偶尔 F.无

A.Frequently B.Always C.Often D.Sometimes E.Seldom F.Never

14.你是否感到疲劳、过累、无力或精疲力竭?

14 Do you feel tired, overtired, weak, or exhausted?

A.所有时间 B.大部分时间 C.很多时间 D.有时 E.偶尔 F.无

A.Frequently B.Always C.Often D.Sometimes E.Seldom F.Never

15.你对自己健康关心或担忧的程度如何?

15 To what extent do you care about your health?

不关心0 1 2 3 4 5 6 7 9 10 非常关心

Never 0 1 2 3 4 5 6 7 9 10 Too much

1. 你感到放松或紧张的程度如何?

16 How relaxed or nervous do you feel?

放松0 1 2 3 4 5 6 8 9 10紧张

Relaxed 0 1 2 3 4 5 6 7 9 10 Nervous

17.你感觉自己的精力、精神和活力如何?

17 How do you feel about your energy, spirit, and vitality?

无精打采0 1 2 3 4 5 6 7 8 9 10精力充沛

Slouching 0 1 2 3 4 5 6 7 9 10 Energetic

18. 你忧郁或快乐的程度如何?

18  How depressed or happy are you?

非常忧郁0 1 2 3 4 5 6 7 8 9 10非常快乐

Depressed 0 1 2 3 4 5 6 7 9 10 Delighted

1. 你是否由于严重的性格、情感、行为或精神问题而感到需要帮助?

19 Do you need help because of a serious personality, emotional, behavioral, or mental problem?

A.是的，曾经寻求帮助 B.是的，但未寻求帮助 C.有严重的问题 D.几乎没有问题 E.没有问题

A.Yes, I once asked for help from others B.Yes, but I have never asked for help from others C.I have serious problems D.I almost have no problem E.I have no problem

20.你是否感到将要精神崩溃或接近于精神崩溃?

20 Do you feel like you are about to have a nervous breakdown or are close to it?

A.是的，在过去的一年里 B.是的，在一年以前 C.无

A.Within one year B.More than one year ago C.Never

21.你是否曾有过精神崩溃?

21 Have you ever had a nervous breakdown?

A.是的，在过去的一年里 B.是的，在一年以前 C.无

A.Within one year B.More than one year ago C.Never

22. 你是否曾因为性格、情感、行为或精神问题在精神病院、综合医院精神病科病房或精神卫生诊所治疗?

22 Have you ever been treated in a psychiatric hospital, general hospital psychiatric ward or mental health clinic for a personality, emotional, behavioral or mental problem?

A.是的，在过去的一年里 B.是的，在一年以前 C.无

A.Within one year B.More than one year ago C.Never

23. 你是否曾因为性格、情感、行为或精神问题求助于精神科医生、心理学家?

23 Have you ever sought help from a psychiatrist or psychologist for a personality, emotional, behavioral, or psychiatric problem?

A.是的，在过去的一年里 B.是的，在一年以前 C.无

A.Within one year B.More than one year ago C.Never

24.你是否曾因为性格、情感、行为或精神问题求助于以下人员? ( 1代表“是”，2代表“否”)

24 Have you ever sought help from one of the following people for a personality, emotional, behavioral, or psychiatric problem? (1 for "yes", 2 for "no")

A.普通医生(真正的躯体疾病或常规检查除外)

A.General practitioner

1 2

B.脑科或神经外科专家

B.Neurosurgeon

1 2

C.护士(一般内科疾病除外)

C.Nurses

1 2

D.律师(常规的法律问题除外)

D.Lawyers

1 2

E.警察(单纯的交通违章除外)

E.Policemen

F.牧师、神父等各种神职人员

F.Priests and clerics

1 2

G.婚姻咨询专家

G.Marriage counselor

1 2

H.社会工作者

H.Social workers

1 2

H.其他正式的帮助

H.Other formal jobs

1 2

1. 你是否曾与家庭成员或朋友谈论自己的问题?

25 Have you ever talked about your problems with a family member or friend?

A.是的，很有帮助 B.是的，有些帮助 C.是的，但没有帮助 D.否，没有人可与之谈论E.否，没有人愿意与我谈论 F.否，不愿与人谈论 G.没有问题

A.Yes, it was very helpful B.Yes, it was helpful C.Yes, but it was unhelpful D.No, there was no one to talk with E.No, there was no one willing to talk with me F.No, I wasn’t willing to talk with others G.I have no problems
